# A Novel 3D Printing Particulate Manufacturing Technology for Encapsulation of Protein Therapeutics: Sprayed Multi Adsorbed-Droplet Reposing Technology (SMART)

**DOI:** 10.3390/bioengineering9110653

**Published:** 2022-11-05

**Authors:** Niloofar Heshmati Aghda, Yu Zhang, Jiawei Wang, Anqi Lu, Amit Raviraj Pillai, Mohammed Maniruzzaman

**Affiliations:** Pharmaceutical Engineering and 3D Printing (PharmE3D) Labs, Division of Molecular Pharmaceutics and Drug Delivery, College of Pharmacy, The University of Texas at Austin, Austin, TX 78705, USA

**Keywords:** protein encapsulation, chitosan nanoparticle, ionic gelation, particle manufacturing, SMART

## Abstract

Recently, various innovative technologies have been developed for the enhanced delivery of biologics as attractive formulation targets including polymeric micro and nanoparticles. Combined with personalized medicine, this area can offer a great opportunity for the improvement of therapeutics efficiency and the treatment outcome. Herein, a novel manufacturing method has been introduced to produce protein-loaded chitosan particles with controlled size. This method is based on an additive manufacturing technology that allows for the designing and production of personalized particulate based therapeutic formulations with a precise control over the shape, size, and potentially the geometry. Sprayed multi adsorbed-droplet reposing technology (SMART) consists of the high-pressure extrusion of an ink with a well determined composition using a pneumatic 3D bioprinting approach and flash freezing the extrudate at the printing bed, optionally followed by freeze drying. In the present study, we attempted to manufacture trypsin-loaded chitosan particles using SMART. The ink and products were thoroughly characterized by dynamic light scattering, rheometer, Scanning Electron Microscopy (SEM), and Fourier Transform Infra-Red (FTIR) and Circular Dichroism (CD) spectroscopy. These characterizations confirmed the shape morphology as well as the protein integrity over the process. Further, the effect of various factors on the production were investigated. Our results showed that the concentration of the carrier, chitosan, and the lyoprotectant concentration as well as the extrusion pressure have a significant effect on the particle size. According to CD spectra, SMART ensured Trypsin’s secondary structure remained intact regardless of the ink composition and pressure. However, our study revealed that the presence of 5% (*w/v*) lyoprotectant is essential to maintain the trypsin’s proteolytic activity. This study demonstrates, for the first time, the viability of SMART as a single-step efficient process to produce biologics-based stable formulations with a precise control over the particulate morphology which can further be expanded across numerous therapeutic modalities including vaccines and cell/gene therapies.

## 1. Introduction

Many industries such as pharmaceuticals, cosmetics, and food are widely using biologics, including proteins, nucleic acids, and even live microorganisms such as bacterial or mammalian cells for many applications [1]. The application of biomacromolecules such as polypeptides, polynucleotides, enzymes, hormones, and antibodies has been growing exponentially in the pharmaceutical industry as novel therapeutic or immunization agents. However, the stability and bioavailability of these drugs has remained a challenge that needs to be addressed [1].

The development of advanced drug delivery systems for the controlled delivery of large biomolecules provides satisfactory solutions for a wide range of encountered issues [2]. Various types of formulations such as organic and inorganic particles have been introduced and extensively studied for the efficient delivery of biologics [3]. Although inorganic materials such as silica nanoparticles (NPs) are demonstrated to be suitable candidates for the delivery of biologics in many research works, especially the proteins, their biocompatibility and biodegradability remains an issue [4]. On the other hand, polymeric particles with both synthetic and natural origins such as poly(lactic acid) (PLA), poly(lactic-co-glycolic acid) (PLGA), polycaprolactone (PCL), and polysaccharides and proteins including but not limited to chitosan, dextran, alginate, hyaluronic acid, albumin, zein, and silk fibroin are alternative carriers that offer both a controlled delivery and a superior biocompatibility and biodegradability [5,6]. Chitosan particles have been gaining extensive attention over the past decades due to their unique feature [7,8,9,10,11]. Owing to its amine and hydroxyl groups, chitosan is an antioxidant agent [12]. It is also shown to be antimicrobial that makes it a great candidate for wounds and implants with a high risk of infection [13,14,15,16]. Chitosan can engulf proteins and polynucleotides upon electrostatic intermolecular interactions, carry them through biological barriers such as the blood–brain barrier (BBB), and sustainedly release the cargo to leave a therapeutic effect without any loss in activity [17,18,19,20,21]. Chitosan particles are also being utilized for enzyme immobilization since they prevent the interference of metal ions with the enzyme’s activity [22,23,24]. In this study, we introduced a facile method to produce chitosan particles for the encapsulation of proteins.

Several methods have been developed for the preparation of chitosan particles. The most common synthesis mechanism includes emulsion- and precipitation-based methods as well as the ionic gelation [25,26]. The necessity of using toxic chemicals such as surfactants and organic solvents that are difficult to remove and might also affect the integrity of the particles payload making the first two classes of synthesis methods less popular compared to the ionic gelation. In this method, the addition of an ionic crosslinker such as sodium tripolyphosphate (TPP) or sodium sulfate to the aqueous solution of chitosan leads to the gel’s formation [27,28]. The gel then undergoes an external stress like an ultrasonication, a homogenization, or a high-pressure extrusion to generate the nano/microparticles [29,30].

The emergence of three-dimensional (3D) printing technology that allows for the designing and production of personalized and patient-specific formulations has an enormous impact on pharmaceutics as well as drug delivery [31,32]. This approach offers a digitalized platform for the production of complex formulations with a desired dosages and structures which leads to an improved therapeutic efficiency and less side effects [33,34]. Several studies have investigated the potential of different types of 3D printers for the production of dosage forms for various therapeutic agents [31,35,36,37,38]. Recently, inkjet 3D printers have been employed to fabricate drug-loaded polymeric particulate systems and appeared as a promising manufacturing approach. For example, paclitaxel-loaded PLGA microparticle, alginate/calcium nano/microparticles, magnetic liposomes, and alginate/carrageenan particles were produced by this platform [39,40,41,42,43,44]. However, none of these approaches offers a provision for the single-step production of stable solid particles with a precise control over the size and morphology of the produced particles alongside the provisions for an integrated flash-freezing platform to produce therapeutics encapsulated micro/nano solid frozen particles. Thus, there is an immense need for an alternative technology in this space.

Herein, we introduced an extrusion-based personalized in vial 3D printing technology, sprayed multi-adsorbed droplet reposing technology (SMART) for the production of protein-loaded chitosan NPs. SMART was originally invented to combine extrusion-based 3D printing and emulsion evaporation or solution dispersion techniques to fabricate novel polymeric micro/nanoparticles. Compared to the conventional emulsion evaporation method, SMART is suitable for the encapsulation of heat-sensitive agents and biomolecules, which employs the shear force exerted by a syringe nozzle rather than the sonication energy to generate microdroplets, eliminating the emulsion or solution cooling step. This platform allows for a rapid production of dry submicron chitosan particles at a controllable dosage and is faster than the traditional technologies. The exertion of extrusion-based printing for making particles that we introduced for the first time offers several advantages. The main benefit of using extrusion-based 3D printing is its compatibility with several mechanisms of particle formation. Furthermore, several inks with a wide range of viscosity can be used in extrusion-based 3D printing, which is a major limitation in inkjet printing. Lastly, extrusion-based 3D printing is the most common method of bioprinting. The incorporation of nanoparticles in a single manufacturing unit can be beneficial to bioprinting for the delivery of several agents, such as the growth factors. As a model protein cargo, trypsin was entrapped in the particles. Further, the product has been thoroughly characterized and the effects of several operational parameters such as the ink’s composition and extrusion pressure were studied.

## 2. Materials and Methods

### 2.1. Materials

The pierce BCA protein assay kit and N-alpha-benzoyl-L-arginine ethyl ester was purchased from Thermo Scientific (Rockford, IL, USA). D(+)-Trehalose dihydrate was obtained from ACROS Organics, (Fair Lawn, NJ, USA), and chitosan ≥ 75% (deacetylated) was obtained from Sigma-Aldrich (CAS: 9012-76-4). Sodium sulfate anhydrous and sucrose and sodium phosphate monobasic monohydrate were purchased from Fisher Scientific (Fair Lawn, NJ, USA). The trypsin from the bovine pancreas and ACS grade acetic acid and hydrochloric acid were obtained from VWR International LLC (Solon, OH, USA).

### 2.2. Ink Preparation

To ensure a consistency of all the prepared batches, the same stock solution of chitosan and sodium sulfate were used to prepare the ink. The deacetylated low molecular weight chitosan was thoroughly dissolved in 0.02% (*v*/*v*) glacial acetic acid by an overnight stirring at room temperature to obtain a 2.4% (*w*/*w*) chitosan stock solution. The trypsin solution was prepared freshly in water prior to the printing process. The ink was prepared by the addition of 15 mg/mL of trypsin, and then 10% (*w/v*) sodium sulfate solutions to the chitosan solution (concentration varied) with/without lyoprotectant at a volume ratio of 1:1:4 (Trypsin: Sodium Sulfate: Chitosan). The ink was incubated at room temperature for 10 min prior to the printing. The ink compositions of all the formulations are listed in Table 1.

### 2.3. Ink Characterization

#### 2.3.1. Particle Size Distribution

Dynamic light scattering (DLS) was used to determine the size distribution of the particles in the ink. The measurement was carried out by a Malvern ZetaSizer Nano ZS instrument at 25 degrees using DI water as the dispersant.

#### 2.3.2. Rheology

The rheological properties of the prepared inks in Section 2.2 were investigated using a Discover HR-2 Hybrid rheometer. All of the measurements were conducted at 25 °C using parallel plates with an 8 mm diameter geometrical setup. The flow curves of each ink, i.e., the shear viscosity versus the shear rate, were obtained over a shear rate range of 0.01 s^−1^ to 300 s^−1^.

### 2.4. Process Setup

**Figure 1:**
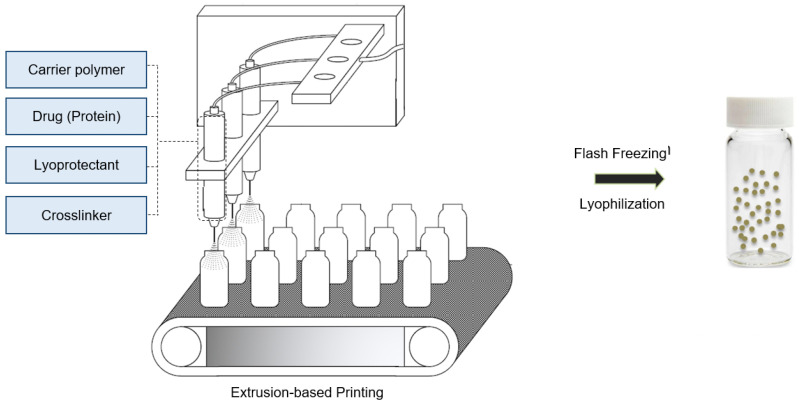
Schematic of Sprayed Multi-Adsorbed Droplet Reposing Technology (SMART).

The trypsin-loaded chitosan particles were prepared using sprayed multi adsorbed-droplet reposing technology (SMART). Briefly, a pneumatic extrusion-based bioprinter (BIOX, Cellink, Goteborg, Sweden) was used to print the prepared inks in Section 2.2 into the vials. An external pump was connected to the printer to allow for a high-pressure extrusion (up to 700 kPa). The printing was carried out using the droplet on demand (DoD) mode at various pressures ranging from 200 kPa to 600 kPa (Table 1 lists the details) using a 25-gauge nozzle with an inner diameter size of 0.437 mm. The vials were immediately placed into a liquid nitrogen bath to flash freeze the particles and they were transferred to a freeze drier for 72 h. Figure 1 shows a scheme of the process.

### 2.5. Product Characterization and Evaluation

#### 2.5.1. Particle Size Distribution

The hydrodynamic size of particles was determined by DLS using a Malvern zeta sizer. The samples were dispersed in DI water prior to the measurement and the measurement was carried out at 25 °C.

#### 2.5.2. Loading Content

A known mass of the powdered product was reconstituted in water. The trypsin concentration of the resulted solution, and consequently the mass of trypsin in each formulation, was measured using a BCA protein assay kit according to the manufacturer’s instructions. The UV-Vis spectroscopy measurements were performed by a Synergy H1 multi-mode plate reader (Biotek Instruments Inc., Santa Clara, CA, USA). The drug loading (%) and loading efficiency was calculated using Equations (1) and (2), respectively.

(1) $$\mathrm{Drug}\;\mathrm{Loading}\;(\%)=100\;\text{×}\;\frac{\mathrm{Mass}\;\mathrm{of}\;\mathrm{drug}\;\left(\mathrm{Trypsin}\right)}{\mathrm{Total}\;\mathrm{mass}\;\mathrm{of}\;\mathrm{powdered}\;\mathrm{SMART}\;\mathrm{product}},$$

(2) $$\mathrm{Loading}\;\mathrm{Efficiency}\;(\%)=100\;\text{×}\;\frac{\mathrm{Measred}\;\mathrm{Drug}\;\mathrm{Loading}\;}{\mathrm{Theoritical}\;\mathrm{Drug}\;\mathrm{Loading}\;},$$

#### 2.5.3. Particle Morphology

The morphology of the produced powder was determined by scanning electron microscopy. The powdered SMART product was deposited on a carbon tape and coated with gold using an ESM sputter coater. FEI Quanta 650 ESEM scanning electron microscopy (SEM) was then used to visualize the microscopic structure of the samples at various magnifications. The operational acceleration voltage was 10 kV at a working distance (WD) of 9.2 mm.

#### 2.5.4. Fourier-Transform Infrared Spectroscopy

An attenuated total reflectance Fourier-transform infrared spectroscopy (ATR-FTIR) on an Infinity Gold FTIR Spectrometer (Thermo Mattson) was utilized to investigate the chemical structure of the powder formulations produced by SMART. To this aim, a powder sample of the microparticles, free drug, and unprocessed chitosan were analyzed. The scanning range was from 400 to 4000 cm^−1^ with a resolution of 64, and the measurement was operated by WINFIRST software.

#### 2.5.5. Circular Dichroism (CD) Spectroscopy 

The powders were suspended in a PBS buffer with a final trypsin concentration of 0.05 mg/mL. Circular Dichroism (CD) spectroscopy was used to investigate the secondary structure of the encapsulated trypsin. A JASCO-810 Spectrometer (Japan) was used for the scanning which was carried out for 200–260 nm wavelengths with a step size of 1 nm at 25 °C. The scanning rate was 100 nm/min and 5 measurements were carried out for obtaining each spectrum.

#### 2.5.6. Enzymatic Activity of Trypsin

Free trypsin and SMART products with an equal concentration of 250 µg/mL were dissolved in cold HCl (0.001 M). N-alpha-benzoyl-L-arginine ethyl ester was dissolved in a sodium phosphate buffer (67 mM) with a concentration of 250 µM. The stock solutions of the enzyme containing the formulations and the substrate were mixed with a volume ratio of 1:15 and were immediately transferred to a Synergy H1 multi-mode plate reader (Biotek Instruments Inc., USA) to read the absorbance of the mixture at 253 nm over a period of 5 min. The relative activity of the enzyme in each sample was calculated using Equation (3).

(3) $$\mathrm{Relative}\;\mathrm{activity}=\frac{\frac{\Delta A_{sample}}{min}-\frac{\Delta A_{blank}}{min}}{0.001},$$

#### 2.5.7. Release Kinetics

The profile of the release kinetics was determined over a 48 h period. A determined mass of the SMART powdered product (120 mg) was thoroughly suspended in 30 mL of 0.1 mM phosphate-buffered saline (PBS, pH 7.4) and aliquoted into 30 individual vials. The vails were incubated at 37 °C while shaking at 100 rpm in an Excella E24R Inc/Ref shaker (New Brunswik, Eppendorf, Hamburg, Germany). Each vial was taken as a test sample at the predetermined time intervals of 0, 2, 4, 6, 8, 10, 24, and 48 h. The samples were collected and filtered using 0.22 µm syringe filters and the trypsin concentration in the filtrates were quantified using a BCA assay kit according to the manufacturer’s instructions.

#### 2.5.8. Statistical Analysis

All the tests were replicated three time (*n* = 3) and the data are reported as the mean ± standard deviation (SD). Single factor analysis of variances (ANOVA) followed by Tukey’s post hoc tests were used to determine the statistical differences between the studied groups (α = 0.05).

## 3. Results

SMART is a particle manufacturing method based on micro-extrusion with a 3D printer which is operated at mild conditions. It is capable of producing particles with a desired size and a high loading capacity, while it is compatible with the delicate loading of molecules that are sensitive to the environmental physiochemical conditions. SMART is an express manufacturing method that can be operated both on-demand and in a continuous mode.

The encapsulation of biologics including proteins, polypeptides, polynucleotides, and cells requires mild conditions to maintain the structure and functionality of the active agent. Therefore, using many common methods such as nanoprecipitation and emulsion-based methods for the preparation of particulate delivery systems are challenging [45]. For example, the presence of an organic solvent might cause a protein denaturation or the disruption of the cell membrane. In this work, we used an ionic gelation mechanism to encapsulate biologics into a polymeric carrier. This method is carried out in an aqueous media. Therefore, the risk of a biologic denaturation or a loss of activity due to the presence of an organic solvent is eliminated. It is also compatible with the conventional 3D printers that are commonly used for bioprinting and can be combined with 3D bioprinting in a single-step process.

Ionic gelation consists of the ionic crosslinking of a water-soluble polymer followed by applying a sufficient force for the disruption of the crosslinked network into smaller identical nano/microparticles [30,45]. If other ingredients such as small or large molecule drugs are present in the solution, they will be entrapped in the crosslinked particles. In this work, chitosan has been used as the model carrier since chitosan NPs have gained extensive attention for the delivery of biologics. Trypsin is encapsulated in the chitosan NPs as a model protein payload. The aqueous mixture of chitosan and trypsin with or without a cryoprotectant was ionically crosslinked by a sodium sulfate. The prepared gel was then extruded using a pneumatic 3D bioprinter to form the final NP product. The properties of the prepared particles and their protein payload are presented.

### 3.1. Ink Characterization

#### 3.1.1. Particle Size Distribution

**Figure 2:**
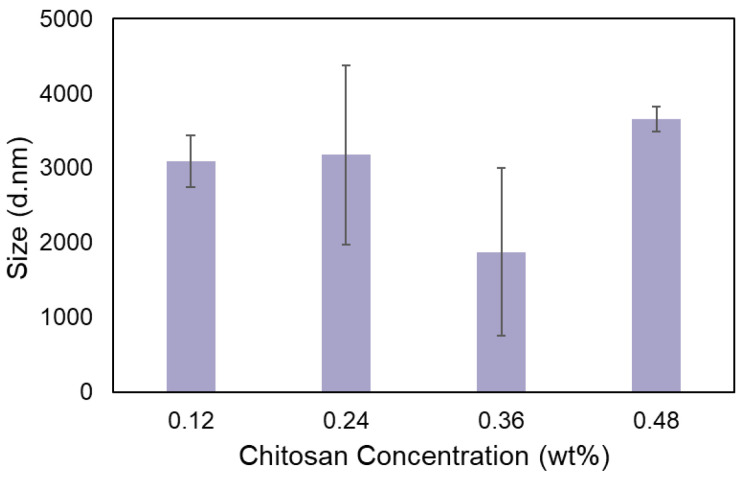
Effect of chitosan concentration on the ink’s average size. Data points represent mean ± SD (*n* = 3).

Various parameters are confirmed to influence the diameter size of chitosan NPs. The chitosan molecular weight, the chitosan concentration in the synthesis media, the pH of the solution, and the mass ratio of the crosslinker and chitosan are the most highly effective parameters [8,46,47]. All the mentioned factors remained constant in our ink formulations except for the chitosan concentration. Thus, the size distribution of the ink formulations with a different chitosan concentration was determined prior to the extrusion process. Figure 2 presents a bar graph that shows the inks average size diameter for the formulations with a different chitosan concentration varying from 0.12% to 0.48% (*w/v*). However, no statistically significant difference is observed as the chitosan concentration increases (*p* = 0.144) as the particle formation process is not completed yet. The average size of the main ink that was used throughout the study (chitosan concentration = 0.24% (*w/v*)) was 3176.7 ± 1201.8 nm with a PDI of 0.82 ± 0.25. It is expected that the pressure-assisted extrusion reduces the particles size to a nanoscale.

#### 3.1.2. Rheology

**Figure 3:**
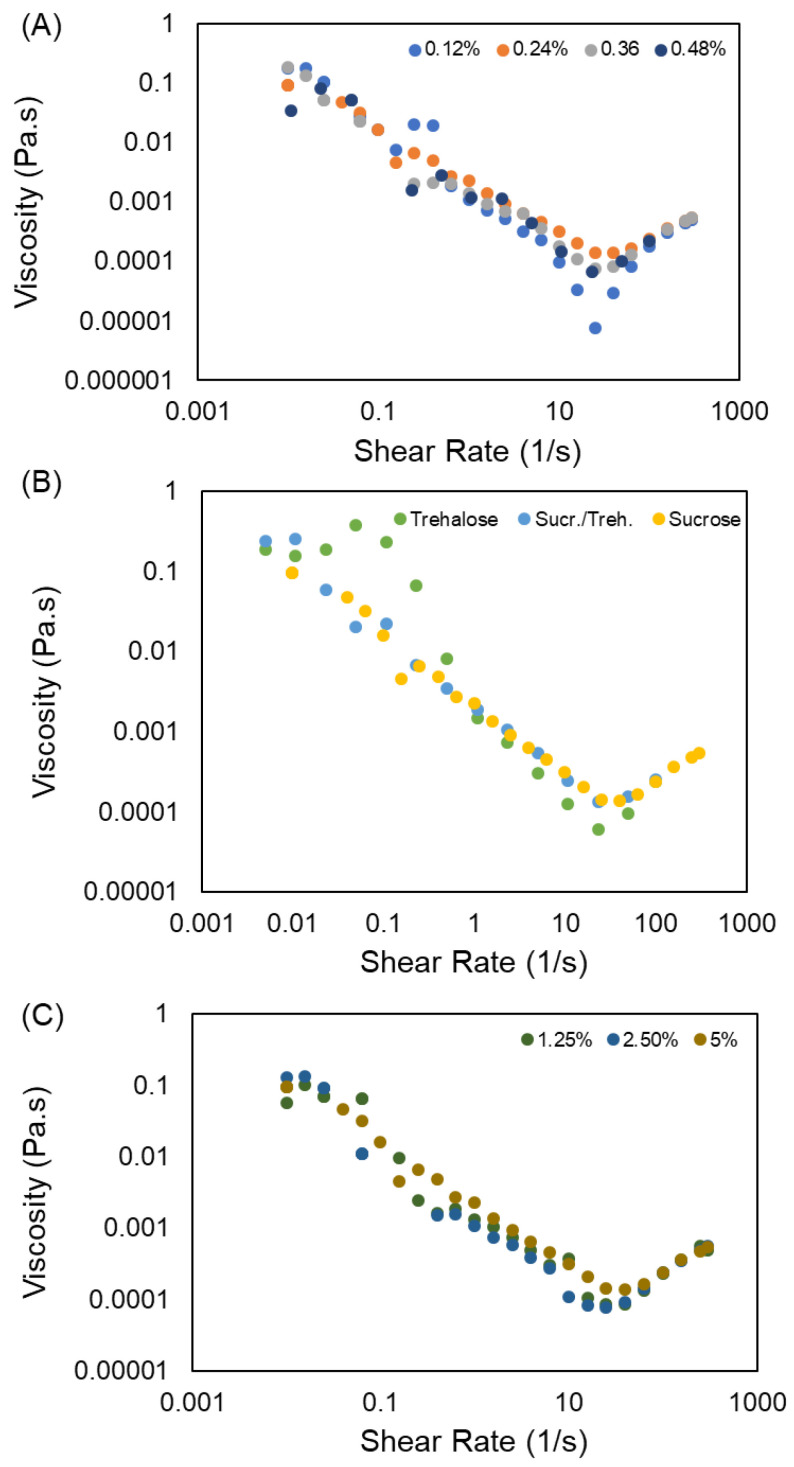
Effect of (**A**) chitosan concentration, (**B**) type of lyoprotectant, and (**C**) concentration of lyoprotectant on flow curves of the inks.

The rheological characteristics of the ink are important features that must be determined prior to the printing process. Figure 3 shows the results obtained from rheological characterization of the inks. The graphs show the viscosity of the inks that are exposed to various shear rates ranging from 0.01 s^−1^ to 300 s^−1^. A very small difference is observed in the viscosity of different ink formulations. Increasing the concentration of lyoprotectant slightly increases the viscosity at all the shear rates below 25 s^−1^ (Figure 3C). Moreover, the same trend is observed for all of the inks. Hence, increasing the shear rate initially decreases the viscosity, showing a shear thinning behavior [48]. However, the viscosity increases again when the shear rate exceeds 25 s^−1^. Decreasing the viscosity is attributed to a disruption of the large, crosslinked gel particles as a response to an exposure to the high stress. Breaking down the crosslinked network results in less resistance and allows for an easier flow. Increasing the viscosity, on the other hand, provides evidence for the formation of structures that resist flowing. This reversible shear thickening behavior of NP dispersion has been reported previously [49,50,51,52]. The reason for this behavior is explained to be a change in the arrangement of NPs when the shear rate increases, changing from a single layer two-dimensional array to a three-dimensional random structure. The particle size distribution, polydispersity, and morphology as well as the concentration of NPs strongly affects this dilatancy behavior. The particles’ diameter size dictates the critical shear rate at which the viscosity starts increasing [53]. As seen in the next section (Section 3.2.1), the diameter size of our product is around 1 μm. Therefore, the observed critical shear rate of 25 s^−1^ is in accordance with other works reported in the literature [53].

### 3.2. Product Characterization and Evaluation

#### 3.2.1. Particle Size Distribution and Drug Loading

**Figure 4:**
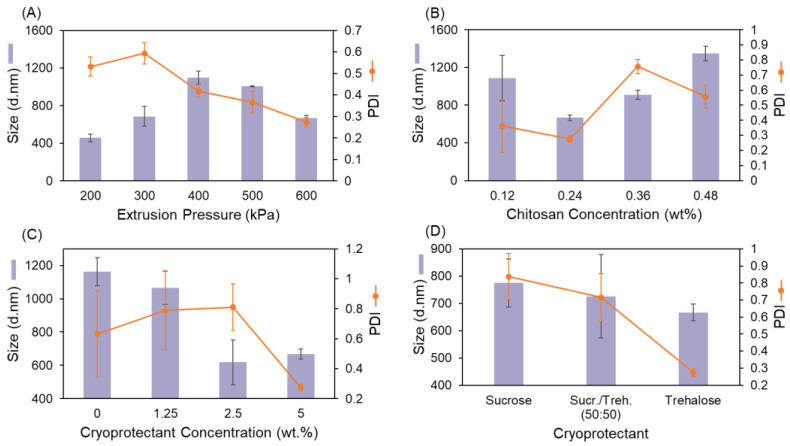
Effect of (**A**) extrusion pressure, (**B**) chitosan concentration, (**C**) trehalose concentration, and (**D**) type of lyoprotectant on product average diameter size and PDI. Data points represent mean ± SD (*n* = 3).

NP size is a crucial feature that plays an important role in the function of NPs. The distribution of the particles in the body, their cell uptake, and the drug release pattern from the particles are some of the most important factors that are highly affected by the NPs size. For this reason, we measured the NP diameter size after processing with SMART and studied the effect of various parameters on the final hydrodynamic size. Figure 4 summarizes the results from this study.

Figure 4A shows a graph that compares the average size and PDI of the formed NPs under various extrusion pressures. An increase in the pressure is expected to increase the applied shear stress and disrupt the formed gels to smaller particles. In our study, as the pressure increases from 400 kPa to 600 kPa, the NP diameter size decreases, as anticipated. However, this trend is reversed in the 200 kPa to 400 kPa pressure range. A possible reason for this observation is that the pressure is this range is not sufficient to break all the particles. The aggregation of unstable broken particles with bigger particles leads to a larger average size. On the other hand, PDI, which indicates the NP size uniformity, decreases continuously as the pressure increases. An extrusion pressure is applied to break the large gel particles into smaller spherical particles. A higher pressure is expected to provide the required shear force to disrupt the large particles to smaller ones. As explained in Section 2.4, in the SMART setup, the working medium is in a 3 mL syringe with a relatively narrow diameter which is connected to a 500 µm sized nozzle at the base. This geometry allows for the homogeneous distribution of the applied stress. As the pressure increases, the stress profile along the cross-section of the nozzle tip become narrower, leading to the formation of a particle with a similar size and a consequently lower PDI. The chitosan concentration is the next factor that we studied to control the NP size. Figure 4B shows the effects of the chitosan concentration on the particles’ size and PDI. A higher polymer concentration results in the formation of particles with a larger mass. In order to gain a stable colloidal system, the higher gravitational force must be compensated by a higher surface tension. Therefore, the result is a larger particle size, which in turn explains the observation in Figure 4B. However, this graph does not show any statistically significant difference between the NP formed by 0.12% and 0.24% (*w/v*) chitosan. Probably because the threshold concentration for the formation and growth of NPs has not been reached and the aggregation of an unsaturated nuclei causes a larger particle size. In the last stage of the SMART process, the formulations undergo a lyophilization, which includes a flash freezing step in liquid nitrogen followed by the sublimation of the water content in a commercial freeze drier. Freezing produces ice crystals that can damage the microscopic structure of the formulation while removing the water, which causes an aggregation, which changes both the size and morphology of the particles. A rapid freezing and the employment of lyoprotectants are the solutions for this issue. Quick freezing does not provide a sufficient time for the ice crystals to grow. According to the heat conduction equation ($\rho c\frac{\partial T}{\partial t}$ = $\frac{\partial^{2}T}{\partial x^{2}}$ + $\frac{\partial^{2}T}{\partial y^{2}}$ + $\frac{\partial^{2}T}{\partial Z^{2}}$ + Q), the freezing time reduces by increasing the temperature gradient as well as decreasing the heat transfer distance, both of which have been applied in the SMART process by using liquid nitrogen as the coolant as well as freezing low-volume samples. We also included lyoprotectants to the ink formulations. Figure 4C,D shows how the presence of lyoprotectant in the formulation affects the particle size. In the absence of lyoprotectant (0%), the particle size was 1162 ± 86 nm. Although including 1.25% (*w/v*) as a lyoprotectant in the ink did not change the size significantly, increasing its concentration to 2.5–5% resulted in a significant size reduction. The highest concentration of Trehalose also resulted in the lowest PDI. As Figure 4D indicates, 5% trehalose leads to the formation of the smallest particles in comparison to the same concentration of sucrose and a mixture of trehalose and sucrose (1:1 mass ratio).

The loading efficiency and drug loading was calculated using Equations (2) and (1), respectively. The average loading efficiency was 91.5 ± 4.6%, which translates to an average drug content of 4.17 ± 0.22% for the formulations that contained 5% (*w/v*) lyoprotectant. Drug loading increased to 11.01%, 8.48%, and 6.28% for the formulations consisting of 0, 1.25%, and 2.5% lyoprotectant due to the lesser amount of the produced powder.

#### 3.2.2. Particle Morphology

**Figure 5:**
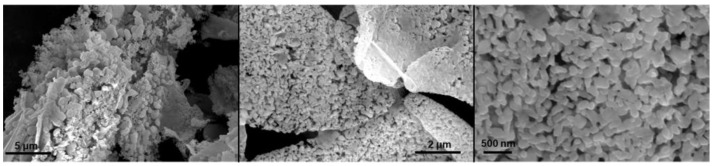
SEM images of powdered product.

Scanning electron microscopy was used to study the morphological microstructure of the powder. Figure 5 shows the representative resulting images. The particulate structure is observed in all of the images. The size distribution of the particles is relatively narrow and acceptable. The size of the particles is smaller than the data reported from the DLS measurement that was discussed in Section 3.2.1. The DLS measures the hydrodynamic size of the particles. Since the particles swell in an aqueous environment, a slight size increase is expected.

#### 3.2.3. Fourier-Transform Infrared Spectroscopy

**Figure 6:**
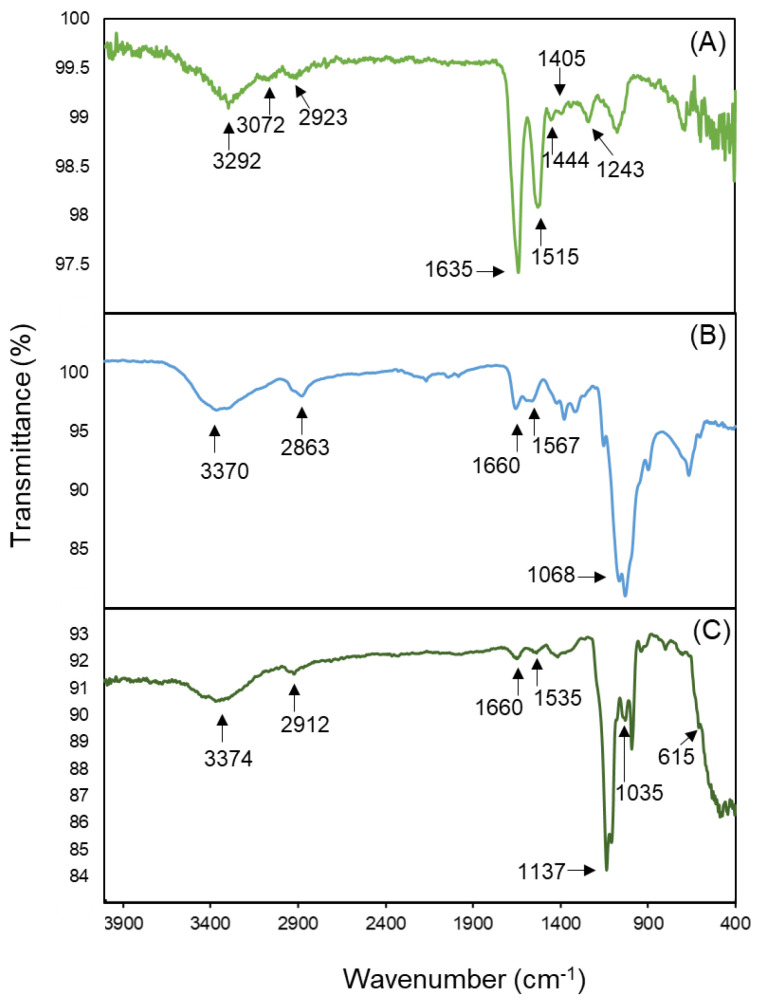
Fourier-transform infrared spectrums of (**A**) trypsin, (**B**) chitosan, and (**C**) trypsin-loaded chitosan particles made by SMART.

The FTIR spectrums of trypsin (A), chitosan (B), and trypsin-loaded chitosan NPs (C) are presented in Figure 6. The main absorption peaks have been marked in all three spectrums. The peaks at 1635, 1243, and 1515 cm^−1^ in Figure 6A, the FTIR spectra of trypsin, are associated with a presence of amides presenting a C=O stretching vibration, –CN stretching vibration, and –NH bending vibration combined with –CN stretching. The peak at 1405 cm^−1^ appeared due to the symmetric stretch of -COO__, while the peaks at 1444, 2923, 3072, and 3292 cm^−1^ indicate a -COH bending, -CH stretching, and –NH stretching vibration of an amid A, respectively [54]. The peaks at 3370 cm^−1^ in Figure 6B belong to the OH and NH groups in the chitosan structure [55]. The same peak is observed in Figure 6C, the IR spectra of the NPs. Similar to trypsin, the presence of amide bonds in the chitosan structure led to the appearance of peaks at 1567 cm^−1^ and 1660 cm^−1^ in Figure 6B and 1660 cm^−1^ and 1535 cm^−1^ in Figure 6C. These two peaks are sharper in chitosan’s spectra. Additionally, two new peaks appeared at 615 cm^−1^ and 1035 cm^−1^ in the particles’ spectrum due to the chitosan ionic crosslinking and the presence of sulfate in the structure [27].

#### 3.2.4. Circular Dichroism (CD) Spectroscopy 

**Figure 7:**
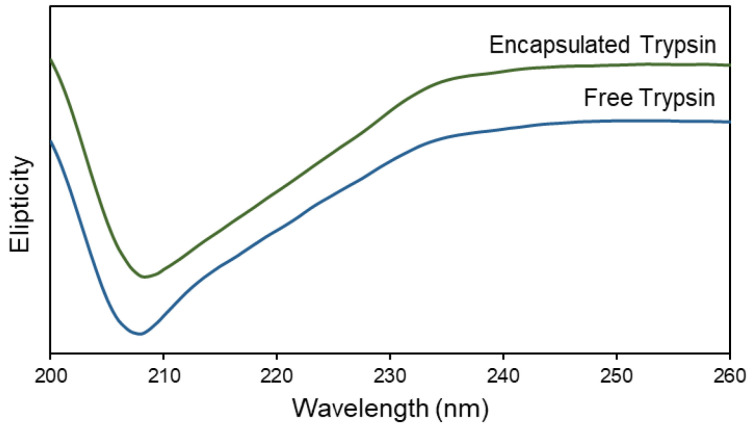
Circular dichroism spectrums of free trypsin and processed and formulated trypsin by SMART.

The biological activity of the proteins is highly impacted by their secondary structure [56,57]. Some operational conditions such as a harsh temperature or a pH change or the presence of specific chemicals may cause conformational changes as well as protein unfolding and, consequently, the loss of activity [58,59,60]. Several studies reported trypsin conformational changes in the presence of environmental conditions such as the presence of salts, various solvents, and temperature [61,62,63,64,65]. We used a far-UV CD spectrum in this work to determine the secondary structure of the encapsulated trypsin and investigate whether the SMART leads to a structural change in the protein. The trypsin concentration was kept consistent in all of the measurements. Figure 7 shows the results from the CD spectroscopy. All of the spectrums appeared similar to the original unprocessed trypsin, confirming that the processing conditions are mild enough to keep the payload structure intact (one representative image has been shown, Formulation 5 in Table 1).

#### 3.2.5. Enzymatic Activity of Trypsin

**Figure 8:**
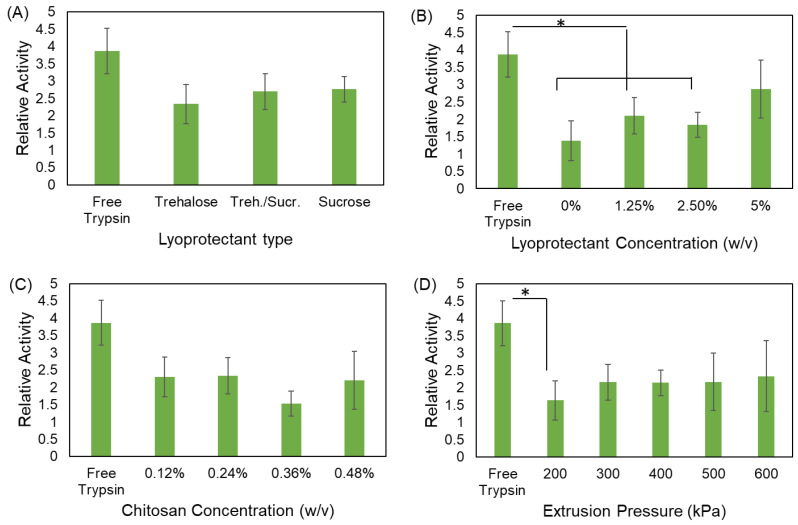
Effect of (**A**) lyoprotectant type, (**B**) lyoprotectant concentration, (**C**) chitosan concentration, and (**D**) extrusion pressure on enzyme activity. Data points represent mean ± SD (*n* = 3). * Significantly different (*p* < 0.05).

Trypsin is an enzyme that digest proteins by catalyzing the hydrolysis of long chain proteins. It breaks large protein molecules into smaller pieces that can further undergo other proteolytic reactions and eventually convert to amino acid block units [66]. Trypsin mostly resides in the small intestine and has a wide range of applications in the food, pharmaceutical, and biotechnology industries [67]. The encapsulation of this protein has been researched for several purposes, including its preservation from degradation and controlled release [68]. However, it is crucial that the encapsulation process maintains the biological activity of the trypsin molecule. In this work, we investigated the effect of several operating factors on the trypsin activity as our model protein to ensure that the SMART is suitable to process the proteins. The reaction of N-alpha-benzoyl-L-arginine ethyl ester with water in the presence of an equal amount of trypsin was observed over a certain time period by monitoring the optical density of the solution at 253 nm, and the relative activity was calculated using Equation (3) [69]. Figure 8 summarizes the results. In comparison to the free unprocessed trypsin, a considerable percentage of activity is preserved, although all of the formulations exhibit a lower activity and most of the differences are not statistically significant. In fact, as depicted in Figure 8D, only when the extrusion pressure was 200 kPa does the trypsin activity significantly reduce, which could be due to the high polydispersity of the particles and the uneven distribution of trypsin throughout the particles. The most significant effect on the trypsin activity was observed when varying the concentration of lyoprotectant in the inks. As shown in Figure 8B, low concentrations of lyoprotectant led to a significant reduction in the trypsin activity. However, the activity of the trypsin in the formulation where the trehalose concentration raised to 5% (*w/v*) is not statistically different from the free trypsin. Therefore, if the ink composition is designed carefully and a sufficient amount of lyoprotectant is included, the enzyme can be preserved biologically active after being processed through SMART, confirming that the SMART platform is a suitable method for protein encapsulation.

#### 3.2.6. Release Kinetics

**Figure 9:**
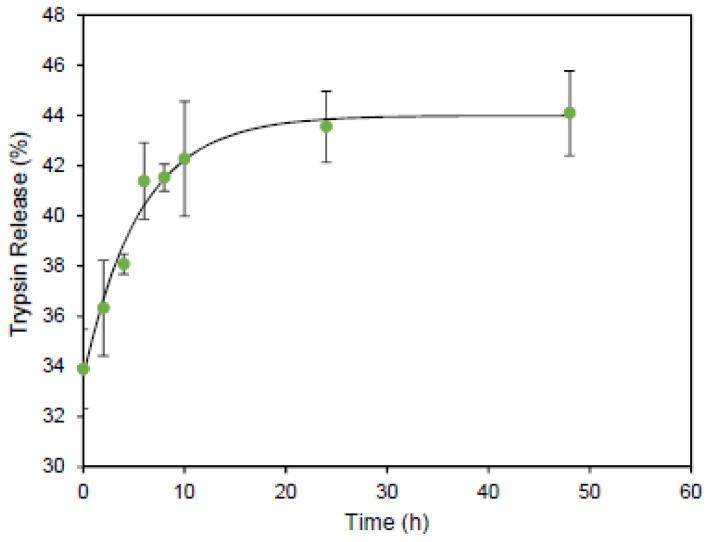
Release kinetics of trypsin from chitosan particles produced by SMART platform. Data points represent mean ± SD (*n* = 3).

Figure 9 shows the profile of the releasing kinetics of trypsin from the chitosan particles. A burst 34% release of trypsin is observed at the initial timepoint, which is associated with the adsorbed trypsin on the surface of the particles that rapidly dissolves in the release medium. However, the rest of the loading content showed a sustained release behavior. The presence of various amino acids with multiple functional groups in the structure of trypsin can undergo various intermolecular interactions with the chitosan carrier particles. These intermolecular forces decrease the rate of protein dissolution by increasing the diffusion coefficient. Therefore, this method is capable of producing particles for a sustained protein delivery. These particles can be beneficial for the pulmonary delivery of proteins. Several studies utilized this administration route for the delivery of free proteins such as insulin since it protects the protein from enzymatic degradation [70,71]. Therefore, the initial burst released drug from chitosan particles will be accessible for a rapid action. On the other hand, in many cases, a prolonged form of the formulation is required to maintain the protein concentration in the blood, which makes the sustained release profile desirable [72,73].

## 4. Discussion

The emersion of therapeutic proteins and their crucial role in the treatment of a wide range of diseases raises a demand for an available, safe, and easy to access and operate manufacturing method that can encapsulate the proteins within the polymeric particles without a significant damaging of, deformation of, or reduction in the biological and therapeutic function of the protein. In this work, we introduced a new method that can successfully generate chitosan NPs encapsulating a model protein trypsin. This method is a simple setup base on a bench 3D bioprinter system that can be accessible even in local developing manufacturing plants, compatible with good manufacturing practice (GMP) regulations since the bioprinter is designed for the aseptic operation and is easy to operate by an operator without extensive training. The dose and size of the product can also be easily adjusted digitally by the 3D printer settings, such as the extrusion time and pressure.

The particle formation mechanism that we chose is water-based and does not require the use of surfactants or polar organic solvents or any other harsh physiochemical environmental condition. Therefore, the protein structure will be preserved from a potential chemical damage. Moreover, the formulations of the inks are tuned to eliminate the need for high tension forces that might damage the protein structure. The SMART system that we used to make the chitosan particles in this work operates at a maximum 600 kPa pressure, which is much lower than the force that an ultrasonication probe or a homogenizer may apply. The force is also applied on the ink while it is flowing through an open syringe at the nozzle tip at a very short residence time, which means the energy does not accumulate. Therefore, it does not cause an ink heating and, consequently, eliminates the need for an extra cooling step. The SMART method is also simple and fast, which becomes important in manufacturing settings where the complexity decreases the production rate and increases the cost of manufacturing.

The formulation dose can be adjusted by the extrusion time to dispense the desired amount of the product. We showed that the particles size can be adjusted by altering the pressure. Thus, besides the dose, we can also tune the size of particles by a digital command in the 3D printer. Another important finding was that the rheological properties of the compositions that we studied did not change significantly by varying the composition. Thus, the printing condition will not be tremendously affected by the ink concentration.

The main parameters that we investigated considering their effect on the smart product properties were the extrusion pressure, chitosan concentration, lyoprotectant concentration, and lyoprotectant type. These findings are important for the adjustment of the properties of the ultimate product, especially for tuning the diameter size. Decreasing the carrier concentration down to 0.24% (*w/v*) and using a sufficient amount of lyoprotectant led to the production of the smallest particles. However, the easiest way to control the size was the adjustment of the extrusion pressure. The higher pressure, that results in smaller particles with a narrow size distribution, is the most favorable.

None of the parameters that we studied, including the high extrusion pressure and various ink compositions, lead to any deformation or unfolding of the protein according to the results from CD spectroscopy. However, the results from testing the trypsin activity confirmed that the ink composition must be carefully designed for maintaining biological function of the protein.

The only parameter that had a significant impact on the functionality and activity of our model protein payload was the presence of a sufficient lyoprotectant in the ink composition. Our results proved that the proteolytic activity of trypsin significantly reduces when we do not include lyoprotectant in the formulation. Although a slight reduction in the activity is observed in other formulations as well, a decent percentage of it is maintained and the difference is not statistically significant.

Another important feature of this technology is that it can be easily converted to a continuous mode where the prepared ink will continuously feed the extrusion chamber (syringe). This will allow the printed formulation to be flash frozen at the printing bed; further allowing the particles to be collected and transferred to a freeze-drying system. Therefore, SMART has a great potential for both a batch and continuous production of protein delivery systems which can be expanded across multiple therapeutic modalities including vaccines.

## 5. Conclusions

According to the importance of protein formulation and their encapsulation for a controlled delivery and enhanced therapeutic effect, in this work we attempted to produce protein-loaded polymeric particles using a novel technology based on pneumatic extrusion-based 3D bioprinting. The model protein and carrier were selected to be trypsin and chitosan, respectively. The novel technology, SMART, that was employed for particle production consists of an initial pressure-assisted extrusion-based 3D printing of the formulation, followed by a flash freezing and lyophilization. Our results proved the concept to be feasible forparticle production without causing damage on the protein by using this first-in-class single-step SMART. We also showed that the particle size and dosage can be controlled and adjusted by increasing the extrusion pressure and time, respectively. In summary, this study demonstrates the use of SMART as an alternative efficient technology for the encapsulation of micro/nanoparticles containing a model protein which may be expanded to the encapsulation of other biologics such as vaccines, polynucleotides and live organisms like bacteria with various functionalities in the future.

## 6. Patents

All authors are co-inventors of related intellectual property (IP) (PCT/US2022/036336).

## Figures and Tables

**Table 1:** Compositions of Ink Formulations.

Formulation	C_Chitosan_ (*w/v*)	Lyoprotectant	C_lyoprotectant_ (*w/v*)	Extrusion Pressure (kPa)
1	0.24%	Trehalose	5%	200
2	0.24%	Trehalose	5%	300
3	0.24%	Trehalose	5%	400
4	0.24%	Trehalose	5%	55
5	0.24%	Trehalose	5%	600
6	0.12%	Trehalose	5%	600
7	0.36%	Trehalose	5%	600
8	0.48%	Trehalose	5%	600
9	0.24%	Trehalose	2.5%	600
10	0.24%	Trehalose	1.25%	600
11	0.24%	Trehalose	0%	600
12	0.24%	Sucrose	5%	600
13	0.24%	Treh./Sucr. (1:1)	5%	600

## Data Availability

The data presented in this study are available on request from the corresponding author.
